# Atrial Fibrillation (AF) in Endurance Athletes: a Complicated Affair

**DOI:** 10.1007/s11936-018-0697-9

**Published:** 2018-10-26

**Authors:** Dimitrios Stergiou, Edward Duncan

**Affiliations:** 1 MSc Sports Cardiology, Cardiology Clinical Academic Group, St George’s, University of London, London, UK; 2 Department of Cardiology, The Bristol Heart Institute, Bristol, UK

**Keywords:** Athletes, Atrial fibrillation, Exercise, Endurance

## Abstract

**Purpose of review:**

A complex relationship exists between exercise and atrial fibrillation (AF). Moderate exercise reduces AF risk whereas intense strenuous exercise has been shown to increase AF burden. It remains unclear at which point exercise may become detrimental. Overall, endurance athletes remain at lower cardiovascular risk and experience fewer strokes. The questions that arise therefore are whether AF is an acceptable byproduct of strenuous exercise, whether athletes who experience AF should be told to reduce exercise volume and how should they be managed. This review aims to critically review the literature and advise on how best to manage athletes with AF.

**Recent findings:**

Emerging evidence suggests that female athletes may exhibit lower risk of AF, but data is limited in female endurance athletes.

**Summary:**

AF is more prevalent in endurance athletes, particularly men and those who competed at a young age. Data is lacking in females and ethnic minorities. Current evidence suggests that treatment options for AF in athletes are similar to those used in the general population; however, medical therapy may be poorly tolerated. Catheter ablation is effective and can allow return to full competition.

## Introduction

Atrial fibrillation (AF) is the most common sustained arrhythmia, affecting at least 1% of the general population [1]. Atrial fibrillation is a leading cause of morbidity and mortality worldwide, with complications including thromboembolic stroke [2], cardiac failure [3], cognitive impairment [4], and a 1.5–1.9-fold increased risk of death independent of cardiovascular disease [5].

A growing list of publications have identified an association between AF and exercise, in particular endurance exercise [6–10]. However, data are not conclusive, controversy continues, and many questions remain unanswered. Notably, although sustained endurance training is associated with an increased AF burden [11–14], moderate exercise is associated with a reduction in AF risk in prospective epidemiological studies [15]. These data are now supported by a recent randomized controlled trial demonstrating a reduction in AF burden in an overweight population through moderate exercise [16]. The 2016 European Society of Cardiology (ESC) guidelines for the management of AF state that “moderate regular physical activity is recommended to prevent AF, while athletes should be counselled that long-lasting intense sports participation can promote AF” (Class of recommendation 1, Level of evidence A) [17]. Hence, the relationship between exercise and AF is complicated.

This review aims to evaluate the current literature, in an attempt to assess at what point does exercise increase the risk of atrial fibrillation and represent too much of a good thing, and whether the negative impact of AF ever outweighs the clear benefits of exercise. Finally, the authors will review treatment options for atrial fibrillation in athletic individuals.

## Methods

An extensive literature search was conducted using the PubMed database up until 2018. The following keywords were used: atrial fibrillation, athlete, exercise, sport, ablation, stroke, CHA_2_DS_2_-VASc. The reference lists of the retrieved articles and the review articles published on the subject were also screened for eligible manuscripts.

### The relationship between exercise and AF

The Cardiovascular Health Study [15] and the Norwegian Tromso survey [18] both demonstrate in a large population that there is a reduction in AF prevalence in those who undertake moderate exercise, but more intense exercise may be associated with increased risk. In keeping with this, further studies suggest that improved cardiorespiratory fitness as measured in METS achieved during exercise testing [19] and VO2 max during cardiopulmonary exercise testing [20] is associated with lower risk of AF at moderate levels. Those with the greatest fitness demonstrate an increased incidence of AF. Importantly, few patients within these populations exercised enough to be described as endurance athletes.

Recently, Calvo et al. have specifically looked at the dose-response relationship between physical activity and lone atrial fibrillation in a case-control study of 157 patients [21]. Care was taken to extend the study into levels of exertion that would include endurance training. Once again, a U-shaped relationship was identified. The authors identify a reduction in AF risk in non-sedentary individuals who complete less than 2000 h of cumulative high-intensity exercise (OR 0.38) and increased risk in those with greater than 2000 h (OR 3.88).

A number of centers have completed studies on highly selected groups of endurance athletes. Anderson et al. examined the largest cohort published [22]. Fifty-two thousand seven hundred fifty-five Swedish long-distance cross-country skiers who completed a 90-km race between 1989 and 1998 were studied and followed up till 2005. Arrhythmia event rates were recorded from national inpatient databases. Those who completed the event quickest or the most times were at the highest risk of atrial fibrillation. This is an important study as it describes a considerably larger cohort than any other, however is limited by a number of assumptions, including that the number of times an athlete competed that particular race and their quickest time relates to their total cumulative lifetime physical activity. The study also lacks a control cohort.

In other observational studies smaller cohorts of endurance athletes have been compared to control groups demonstrating increased risk of AF in endurance skiers [11, 14], cyclists [23], and runners [24, 25]. These studies did include control cohorts, were more thorough in confirming the diagnosis of AF, carefully documented exercise levels through questionnaires, but out of necessity involved smaller numbers of subjects.

Importantly, in keeping with the reduction in AF previously associated with mild or modest exercise, non-endurance sports are not associated with increased risk of arrhythmia, e.g., golf [23] and handball [26].

With the number of cohorts presenting increased risk of AF with endurance sport, it might be expected that meta-analyses of these data would clearly support endurance exercise as a risk factor for AF. This is not however the case, and it is the source of controversy.

Three meta-analyses have been published on this subject. The first in 2009 included 6 studies [27] all of which compared athletes to a control cohort. The athlete cohorts were young (mean age 51) and predominantly male (93%). Athletes were found to be at 5-fold increased risk of AF.

The second meta-analysis published in 2014 included 19 studies and 511,503 participants [28]. Importantly, this study examined the relationship between physical activity and AF over a range of intensities of exercise. No significant relationship was identified between moderate and intense physical activity and AF; however, a subanalysis of athletes did identify increased risk in the sportsmen (HR 1.98). This meta-analysis highlights the importance of the U-shaped relationship between exercise and AF, where moderate exercise appears to reduce AF risk, whereas intense endurance sport and a sedentary lifestyle are associated with greater risk. Thus, the point of the curve that one chooses to study will impact significantly on the relationship identified between exercise and AF.

The third meta-analysis published in 2018 identified six cohort studies and two case-control studies totaling 9113 individuals. Athletes were at increased risk of AF (odds ratio 1.64); however, when adjusted for age, this effect was only identified in those < 54 years of age [29]. Older athletes were not at increased risk of AF compared to age-matched sedentary controls.

### Predictors of AF in endurance athletes

Left atrial volume is significantly greater in endurance athletes compared to strength-trained athletes and the normal population [30–32], and has been identified as a predictor of AF in athletes [11, 33]. Pellicia and colleagues demonstrated in a cohort of 1777 athletes from a mixture of sports that 20% show significant left atrial (LA) dilatation. Dilatation was more commonly seen in endurance sportsmen (rowing, cycling) than in other sports (football, rugby), and a greater proportion of athletes with dilated atria had competed at international level compared to those with normal chamber size. Greater structural adaptation to exercise (i.e., greater left ventricular end-diastolic volume and mass) was also noted to predict LA dilation. This study identified a very low risk of atrial arrhythmia in the cohort; however, notably the athletes were very young (24 ± 6 years) and duration of endurance training was therefore relatively short. In contrast, Grimsmo et al. identified an incidence of AF of 12.8% after 28–30 years of follow-up in cross-country skiers [11]. A dose response to endurance training has also been described. Elliot et al. reported LA dilatation in 19%, 12%, and 0% of athletes who had competed > 6000, 3000–6000, and < 3000 cumulative hours of endurance training [34].

A low heart rate in normal healthy men has been shown to be a risk factor for future AF [35]. Athletes have lower resting heart rates and higher vagal tone compared to controls [36] and heart rate is often used as a surrogate for vagal activity. During 28–30 years of follow-up, bradycardia at rest was the sole predictor of AF in Norwegian cross-country skiers [37]. Similarly, during 20 years of follow-up, the Tromso survey of 20,484 adults identified increased risk of AF as heart rate decreased [18]. This study also highlighted the U-shaped curve between cumulative physical activity and AF. Those who did the most vigorous activity had the lowest heart rate and the highest risk of AF. Interestingly, when heart rate variability was used as a parameter of vagal activity (rather than heart rate itself), no correlation between cumulative hours of training and vagal tone was identified [34].

Cumulative duration of high-intensity endurance training predicts AF. This has been clearly established by a number of studies as endurance exercise has often been measured in terms of total lifetime physical activity rather than amount of exercise per unit of time. The published cutoff values used to differentiate risk are 1500–2000 h total of intense exertion [21, 33].

### Gender and risk of AF in athletes (Fig. 1)

The role of gender is unclear. Greater than 90% of athletes included in the existing literature are male; hence, there is a lack of data relating to risk of AF in female endurance athletes. Subgroup analysis of most datasets is not possible as they are largely underpowered. The Tromso study from Norway is the only study that included a large female cohort [18]. Ten thousand one hundred eighty-four women were followed for 20 years demonstrating a similar U-shaped curve to that seen in men when correlating AF risk with cumulative exercise. Female endurance athletes appeared at the same risk of AF as sedentary women. Also, AF risk increased at lower intensities of exercise than in the male cohort. Notably, however, this was a large study of the general population, was not studying athletes specifically, and has been contradicted by other work. Wilhelm et al. [38] examined middle-aged non-elite athletes (equally represented by female and male athletes) and demonstrated a 6.6% prevalence of AF in men, whereas no women developed AF. Everett et al., who exclusively included women, showed that in women who achieved 7.5MET-h/week of physical activity, the risk of AF was lower compared to those who did not [39]. Finally, a meta-analysis from Mohanty et al. reported that among women the risk of AF displayed a decreasing trajectory with increased level of physical activity [40]. It must be stressed, though, that the level of physical activities defined as “intense” in this study included ≥ 4 h of exercise per week, heavy physical workload, endurance exercise 3 times per week for < 20 years or 20–39 years, and activities requiring ≥ 6 METs-hours/week (jogging, running, aerobic exercise or dance, racquet sports, lap swimming). These levels of exercise are well below what one would expect in competitive endurance athletes, and nowadays, women who participate in endurance competitions surpass in terms of performance women included in the aforementioned studies. To date, most data available suggests that increased exercise reduces AF risk in women. But there remains a lack of data on high-level female endurance athletes.

**Fig. 1:**
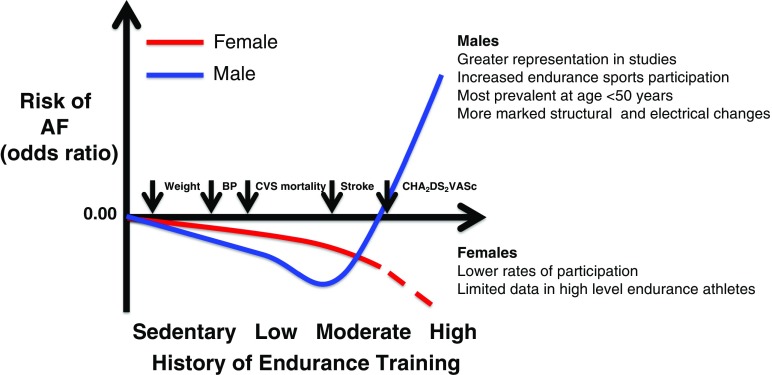
Schematic of AF risk in males and females according to levels of participation in endurance sports. Male and female athletes show different risk profiles for AF as levels of endurance exercise increase. High-intensity endurance training is associated with increased risk of AF in males. The reverse is seen in females, although less data is available in females (dashed line). Despite an increasing AF burden in male endurance athletes, weight, blood pressure (BP), cardiovascular (CVS) mortality, stroke, and CHA_2_DS_2_-VASc scores fall in this group. Figure modified from Mohanty et al [40].

Few data exist regarding predictors of AF in athletic females compared to males; however, in runners completing a 10-mi running race, female athletes demonstrated shorter P wave duration, less left atrial dilatation, and lower vagal tone compared to male counterparts, in keeping with the lower AF risk described above [38].

### The pathogenesis of AF in endurance athletes

A number of hypotheses have been proposed to explain the increase in AF seen in endurance athletes; however, further research is needed.

Adverse atrial electrical and mechanical remodeling may promote AF. Brugger et al. stratified male athletes into low (< 1500 h)-, intermediate (1500–4500 h)-, and high (> 4500 h)-intensity training [41]. High-intensity training was associated with LA dilatation and increased P wave duration, both of which have been linked to the pathogenesis of AF. Echocardiographic measures of left atrial wall strain were also elevated suggesting increased atrial stretch during intense exercise as a potential mechanism. Similar left atrial adaptation has been shown to occur in parallel to increased parasympathetic tone and atrial ectopic activity in marathon runners [37]. The same group later showed that pro-atrial natriuretic peptide (pro-ANP) is elevated in runners on completion of a marathon [32]. Pro-ANP is released upon atrial stretch.

Increased vagal tone in endurance athletes may increase dispersion of atrial repolarization through shortening of the atrial refractory period. To date, increased vagal tone has been associated with AF in athletes and certainly many athletes describe a vagal pattern of AF. However, the mechanism linking the two requires further study.

Ectopy from the pulmonary veins seems to be the predominant trigger for paroxysmal AF [42]. Wilhelm et al. showed that in middle-aged non-elite runners, premature atrial beats were increased in relation with the number of marathons completed and the accumulated training hours [32, 38]. On the other hand, this was not the case in former professional cyclists who did not show any differences regarding premature atrial beats, when they were compared with age-matched golfers [23].

Additionally, large increases in diastolic pulmonary pressures during endurance exercise were recorded by Claessen et al. [43], suggesting remarkable elevation in left atrial pressures. Significantly elevated pressures in the left atrium during prolonged endurance training may explain atrial enlargement seen in highly trained athletes [37, 38, 44]. If exercise stress continues without sufficient time for recovery, this could lead, in some people, to inflammation and fibrosis, thus creating a potential substrate for arrhythmias [45]. However, measurements of left atrial dimensions and volume are not sufficient to provide information about the function of the left atrial cavity. This was supported by Brugger et al. who reported that in 95 amateur male runners older than 30 years, left atrial anatomical and electrical remodeling is not related to atrial function [41]. Importantly, however, when atrial function is analyzed by 2D strain echocardiography, reduced atrial function is strongly associated with paroxysmal AF [46].

Atrial fibrosis has been directly observed in one experimental study by Benito et al. in male Wistar rats [49]. Importantly, fibrotic changes were readily reversed by exercise cessation. Reversal of fibrosis has not been demonstrated in humans. A study by Lindsay et al. did find increased pro-fibrotic markers in 45 elite veteran athletes. When compared with sedentary controls, these athletes displayed higher levels of three biomarkers of cardiac fibrosis, namely, plasma carboxyterminal propeptide of collagen type I (PICP), carboxyterminal telopeptide of collagen type I (CITP), and tissue inhibitor of matrix metalloproteinase type I (TIMP-1). The authors suggested that fibrosis occurs as part of the hypertrophic process in endurance training [47]. D’Ascenzi et al. applied new echocardiographic techniques to indirectly evaluate left atrial fibrosis by estimating myocardial stiffness, which in turn is directly related to the amount of fibrosis. The results were normal or even lower than normal in the left and right atrium of athletes compared with the sedentary individuals and did not show any variation in response to exercise [48].

Inflammatory cytokines release after strenuous exertion has been hypothesized as a mechanism via which exertion causes atrial remodeling and in turn propagation of AF. Runners completing a Swiss mountain marathon have elevated pro-inflammatory cytokines, highly sensitive CRP and leukocytes while showing temporary prolongation of signal averaged p wave duration as a marker of atrial conduction delay [31].

### The clinical significance of AF in endurance athletes

Exercise is associated with a reduction in all cause cardiovascular mortality and stroke [50]. Sedentary patients appear to benefit most from a small increment in exercise. However, even endurance athletes demonstrate reduced risk with increased training, although this is controversial because endurance athletes have previously been under-represented in observational studies. Kettunen et al. did however study 2363 elite athletes (both endurance and power sports) and 1657 controls with a median follow-up of 50 years [51]. In this cohort, the endurance athletes demonstrated reduced total mortality (HR = 0.70), ischemic heart disease mortality (HR = 0.68), and stroke mortality (HR = 0.52). Hence, athletes appear to not die of stroke despite the increased incidence of AF. A likely explanation is the reduction in conventional risk factors as exercise increases from nothing through to intense physical activity. The EORP-AF registry followed 2442 patients diagnosed with AF [50]. Eighty-one percent of the cohort had a CHA_2_DS_2_-VASc score > 2. Patients were categorized as sedentary or taking limited exercise, moderate exercise, or intense exercise. As exercise increased through the groups, CHA_2_DS_2_-VASc score reduced and fewer patients in the high-intensity group had a score > 2 requiring anticoagulation. The high-intensity exercise group also reported lower rates of coronary artery disease, heart failure, and stroke. Interestingly, though, the high-intensity group had lower total mortality regardless of age, gender, type of AF, and CHA_2_DS_2_-VASc score. This suggests that the lower CHA_2_DS_2_-VASc score does not confer all the risk reduction.

The high-intensity exercise AF group also showed lower rates of progression from paroxysmal to persistent and permanent AF than the more sedentary groups [50]. AF type has not however been shown convincingly to influence stroke risk.

Interestingly, endurance athletes with AF report more adverse symptoms than sedentary patients [50]. Despite this, veteran athletes with AF maintain greater levels of activity than non-athletes with AF [52].

In the general population, stroke risk is best estimated using the CHA_2_DS_2_-VASc score. This scoring system has not been validated in athletes, which therefore represents an important area of future study. It is however the best tool available to clinicians at this time.

### Treatment of AF in endurance athletes (Table 1)

Non-athlete patients with AF are offered rate or rhythm control according to symptoms, type of AF (paroxysmal vs persistent), and patient choice [17]. Few data of good quality exists comparing these different approaches in athletes [53–55]. Athletes with paroxysmal AF suffer reduced performance. Unfortunately, pharmacological options are limited. Beta-blockers are often poorly tolerated. Sotalol and amiodarone are not attractive to this cohort of patients due to their side-effect profiles [56]. Flecainide has been associated with exercise induced broad complex tachycardias, syncope, and 1:1 conduction of atrial arrhythmia such as typical flutter [57–60]. International guidelines recommend that flecainide should be prescribed alongside a beta-blocker and athletes taking flecainide for AF should refrain from sports as long as AF persists and until two half-lives of the antiarrhythmic drug have elapsed [17].

**Table 1:** Summary of key treatment options available to athletes with atrial fibrillation

Treatment options in athletes with AF	Advantages	Disadvantages	Comment
Anticoagulation (CHA_2_DS_2_-VASc ≥ 2)	Reduces stroke risk	CHA_2_DS_2_VASc not validated in athletes	There is no evidence that stroke risk in athletes and non-athletes with the same CHA_2_DS_2_-VASc score are the same
Flecainide	Reduces frequency and/or duration of AF episodes	Should be prescribed with a beta-blocker (see below)	ESC guidelines recommend no sporting activity until 2 half-lives of flecainide have elapsed due to pro-arrhythmic properties and risk of rapidly conducted flutter
Beta-blockers	May reduce AF burden in isolation or alongside flecainide	Reduced performance Poorly tolerated in setting of sinus bradycardia	Athletes are generally intolerant of or unwilling to take beta-blockers
Catheter ablation	May eradicate AF allowing return to full competition	Risk of complications May require multiple procedures	Most popular with athletes. Athletes dislike taking medication and look for a permanent fix

De-training has been shown to reduce AF susceptibility in endurance exercise rat models [12]. Importantly, atrial dilatation and fibrosis failed to resolve with this approach. Despite the lack of data in humans, some physicians advocate de-training to reduce AF burden in athletes. This approach requires further study, but it is generally unacceptable to the athletes themselves.

Catheter ablation should be considered in athletes with AF and is often the treatment that the athletes prefer [17]. Results of catheter ablation in small non-randomized cohorts of patients have been described [53–55] and outcomes are reported to be similar to those achieved in non-athletes. Importantly, no data is available on ablation outcomes in athletes with persistent atrial fibrillation or structurally abnormal hearts, e.g., significant atrial dilation or left ventricular dysfunction. Catheter ablation allows the athlete to return to competition without ongoing antiarrhythmic drug use.

## Discussion

Current literature suggests that moderate levels of exercise reduce the risk of AF in all individuals. Male endurance athletes appear to be at increased risk of AF, but they are at lower risk of cardiovascular events, and in particular stroke, possibly due to the overall lower cardiovascular risk profile conferred by improved fitness. Hence, the increased risk of AF should not always be used to argue for dramatic reduction in exercise in this cohort.

Studying AF risk in athletes is challenging due to the heterogeneity of the different endurance sports, the need for long-term follow-up, the variable exercise performed by individuals during follow-up, and the large cohorts required to give adequate power to the study. Furthermore, there is a complex relationship between cumulative exercise performed and risk of AF. Most studies also depend heavily on patient questionnaires to estimate cumulative physical activity and to identify athletes with AF. Alternatively, national databases can be examined for the diagnosis of AF but this relies heavily on the quality of the data recorded. Another source of bias is that athletes are reportedly more symptomatic from AF than sedentary individuals, and also represent a highly motivated group, therefore may be more likely to be diagnosed. Despite these issues, there appears to be a consensus that endurance athletes experience more AF than those engaging in moderate levels of exercise.

In the context of AF, it is vital that the athletic cohorts studied are truly representative of top level endurance athletes with comparable training regimes. Indeed, careful description of exercise intensity in any population studied is important to allow data to be accurately interpreted. Currently, there is a notable lack of data about AF risk in female endurance athletes and athletes from ethnic minorities. It is important to gain insight into these groups in the future. The existing literature, however, does not suggest an increased risk of AF in females engaging in at least moderate levels of exercise.

Importantly, unlike in the general population, AF might not confer as great a risk of stroke and cardiovascular mortality in athletes. This relationship is very important and requires further elucidation. In particular, the CHA_2_DS_2_-VASc score needs to be validated in athletes. Physicians lack evidence of how to specifically manage AF in athletes and therefore do so according to the same principles as used in the general population. Athletes though are unlikely to de-train or opt for medical management with antiarrhythmic drugs and generally have a lower threshold for accepting catheter ablation.

AF diagnostics are also likely to be entering a new era. It has been shown already that increased duration of monitoring results in a dramatic rise in AF diagnoses. This has been emphasized by recent studies in patients suffering cryptogenic stroke in which the use of implantable loop recorders has dramatically increased the diagnostic yield of AF [61]. New “ECG patches” are ideal for prolonged Holter monitoring in sportsmen. The public are investing in personal heart rate and activity monitors. Governments are considering nationwide AF screening programs to reduce stroke risk. These innovations will all increase the number of AF cases identified and can be expected to give further information about AF in athletes compared to the normal population.

Finally, studies need to be performed that will guide sedentary subjects and athletes to reduce their AF burden. Optimal exercise programs that reduce AF risk alongside overall cardiovascular risk are needed for the sedentary group. Clarification of the role of de-training athletes is required as are further studies examining the role of catheter ablation in this group. The overall target must remain the reduction of stroke risk and overall cardiovascular mortality. Fortunately, athletes remain at lower risk than sedentary controls despite their predisposition to arrhythmia.
